# Reliability and Validity of the Turkish Version of the Gross Motor Function Measurement (GMFM-88&66) in Children with Cerebral Palsy

**DOI:** 10.3390/children11091076

**Published:** 2024-09-02

**Authors:** Tuğçe Ataç, Cemil Özal, Mintaze Kerem Günel

**Affiliations:** 1 Institute of Health Sciences, Hacettepe University, Ankara 06100, Turkey; atactugce22@gmail.com; 2 Faculty of Physical Therapy and Rehabilitation, Hacettepe University, Ankara 06100, Turkey; mintaze@hacettepe.edu.tr

**Keywords:** cerebral palsy, gross motor function, psychometrics, reliability, validity

## Abstract

Background: The gross motor function measurement is considered as the gold standard for the motor assessment of children with cerebral palsy. The aim was to carry out the cross-cultural adaptation and investigate psychometric properties. Methods: A total of 150 children with cerebral palsy aged 2–16 (mean 8.82 ± 3.78 years; 54.7% male) included. The Gross Motor Function Measurement was adapted into Turkish. Two physiotherapies independently administered the gross motor function measurement. Internal consistency and intra/inter-rater reliability were assessed using Cronbach’s alpha, intraclass-correlation-coefficient. Standard-error-of-measurement, minimal-detectible-change calculated. The Bland–Altman method was applied to estimate the measurement bias in reliability analysis. Construct validity assessed with Spearman’s correlation coefficient between the gross motor function measurement and the gross motor function classification system, pediatric-evaluation-of-disability-inventory—mobility; confirmatory-factor-analysis was carried. Results: Internal-consistency (α: 0.997–1.00); reliability indices were excellent for total scale (intraclass-correlation-coefficient for intra-rater reliability 0.994–0.999, inter-rater reliability 0.997–0.999) and for each sub-dimension and total score. Standard-error-of-measurement was ranging 1.044–1.677, minimal-detectible-change was 2.435–5.520. Construct validity was supported by strong to excellent negative significant correlations (*p* < 0.05).

## 1. Introduction

The most frequent cause of physical disability in children is cerebral palsy (CP) [1], presented by motor function, posture, manual ability, and communication impairments [2], and may result from activity limitations such as mobility limitations [3]. The main activity limitation related to CP includes motor dysfunction [4] that negatively affects children daily living activities, self-care activities, and social communication [5].

Motor function development is necessary for the achievement of skills and providing children with domestic and social participation as school and leisure. Establishing routines of physical activity that last a lifelong is also crucial for a healthy development into adulthood [6]. Therefore, mostly, the modalities aimed primarily on motor outcomes related to the activity domain [7]. Precise motor function evaluation provides a base to set goals in clinical rehabilitation practices for children with CP. Evaluating motor functionality with standardized measures gives information about individual abilities in different contexts [8], as well as the medical and surgical treatments and rehabilitation practices to be applied, and in creating evidence in research due to the influence of gross-fine-oral motor development [9]. Although there are several tools to assess motor function, the GMFM, proposed by Russell et al. (1989), is a criterion-referenced assessment tool to detect changes in gross motor functionality during the treatment procedure in children with CP [10], and today it is accepted as the “gold standard” of motor function evaluation in CP. It is commonly used for clinical practice as well as research purposes because of the strong psychometric characteristics of the measure, including its responsiveness to change [8,9]. Moreover, it has been validated in different populations with motor impairment, including acquired brain injury, spinal muscular dystrophy, osteogenesis imperfecta, acute lymphoblastic leukemia, and down syndrome [11]. The GMFM translated into several languages, including Brazilian Portuguese [12], Korean [13], Persian [5], and recently Spanish in Spain and Colombia [14,15]. However, although it has been widely used in different cultures so far, its translation into Turkish and its validity and reliability have not been demonstrated. On the other hand, it is important to use the measure in clinical settings in the main language of children with CP to evaluate accuretly, as well as for research purposes since the measure is the gold standard for evaluating motor function. The evidence of validity and reliability are prerequisites to assure the integrity and quality of a measurement instrument; therefore, the aim of this study was to carry out a cross-cultural adaptation of the original GMFM for use with Turkish-speaking children and to investigate the psychometric properties of the Turkish version of GMFM.

## 2. Materials and Methods

The study was a methodologic observational study of the psychometric properties of the Turkish version of GMFM, conducted between February and November 2019 in Hacettepe University, Faculty of Physical Therapy and Rehabilitation, Cerebral Palsy and Pediatric Rehabilitation Unit.

### 2.1. Subjects

Sample size determined according to the consensus-based standards for the selection of health status measurement instruments (COSMIN) checklist for assessing the methodological quality of studies on measurement properties of health status measurement instruments and sample size recommendations for studies on reliability and measurement error [16,17]. In accordance with this guideline, a total of 150 children with CP, without any concomitant conditions, had severe vision and/or hearing problem, and severe intellectual problems included.

The inclusion criteria were ages between 5 and 18 years and being able to follow instructions for the test; the exclusion criteria were having severe cognitive impairment that constrains understanding of test instructions, visual system and/or hearing problems (e.g., severe visual impairment), children with concomitant conditions, The ethical approval was gathered from the non-interventional clinical research ethical committee of Hacettepe University University (No: GO 19/106), performed according to the Helsinki Declaration. Parents of all children signed written informed consent.

### 2.2. Outcome Measures

Gross Motor Function Measure (GMFM): GMFM consists of 5 domains: A-lying-rolling, B-sitting, C-crawling-kneeling, D-standing-walking, and E-running-jumping. The scores are ordinal on a 4-point grade scale after observing the movements. The scores are ordinal on a 4-point grade scale after observing the movements. In this study, 88 and 66 versions were used. The GMFM-66 version was developed by extracting 66-items of GMFM-88 after undertaking Rasch analyses. After completing the test, the scores of all items are plotted using the Gross Motor Ability Estimator (GMAE) software version 2, which converts the result into a scale ranging from 0–100 [18].

Gross Motor Function Classification System (GMFCS): The purpose of GMFCS is to determine the differences in level of motor functionality in children with CP as well as to evaluate validity. The GMFCS classifies the motor impairment within five levels. The distinction between levels is organized according to the functional limitations, the necessity for mobility aids using by hand as walker and crutches or wheeled mobility devices, and to a lesser extent, the quality of the movement. The higher level indicates more severe functional deterioration [19]. In Turkish GMFCS test-retest reliability found high (ICC: 0.94; 95%CI: 0.94–0.98) [20].

Pediatric Evaluation of Disability Inventory—Mobility (PEDI-Mobility): The PEDI-Mobility, based on some aspects of mobility performance, consists of 59 items. These items were rated 0 or 1 based on capability of performance. A higher score expresses better mobility functionality. The Turkish version of the PEDI is found to be valid (Spearman correlation coefficients ≥ 0.86) and reliable (Cronbach’s alpha ≥ 0.98 and ICC ≥ 0.96) [21].

### 2.3. Procedures

CP clinical subtypes were classified according to Surveillance of Cerebral Palsy in Europe and classified as spastic, dyskinetic, ataxic, and non-classifiable [22]. In the initial assessment, parents of participants completed the PEDI-Mobility.

Validity and reliability procedure: Validity and reliability procedures were applied according to the COSMIN guideline [17].

Reliability: Two physiotherapists, experienced at least ten years in pediatric rehabilitation and certificated and experienced in using the GMFM, participated in the study as raters. One physiotherapist (the third author) with at least 30 years of experience in rehabilitation of children and certificated and experienced in using the GMFM tested all children in a quiet room. All items tested by an order according to the user’s manual of GMFM-88 and GMFM-66 [18]. The testing was recorded by video camera. For study of the inter-rater reliability, a total of 150 video recordings were independently viewed by 2 raters. In the baseline assessment, these raters were not involved in the recording of videos. One of the 2 raters (TA) viewed 66 video recordings twice for the intra-rater reliability part of the study. Every third video record randomly selected for intra-rater assessments, and to avoid recall bias, at least after 4 weeks from the first assessment video-record viewed.

Concurrent validity: Two physiotherapists (TA, CO) conducted interviews with parents face-to-face to assess their children’s mobility skills using PEDI-Mobility.

### 2.4. Translation and Cross Cultural Adaptation Process

Written permission was gathered from CanChild Centre, which possesses the publication rights of the original scale. The translation process and adaptation were carried out according to the COSMIN checklist, translation guidelines for translation, and cross-cultural adaptation of outcome measures with the following stages [17]:

(a) Forward translations into the Turkish by independently working two therapists who both have a mother tongue in the Turkish, have experienced at least ten years in pediatrics rehabilitation, and are certificated GMFM users; (b) reconciliation; (c) back-translations: two translators working independently to back-translate into English; (d) overview and discussion of the back-translated version; (e) proofreading by an expert translator hired by the developer; (f) pilot test on a group of children with CP; (g) review of the pilot test on children with CP by the expert panel, including a pediatric neurologist, a developmental pediatrician, a general pediatrician, a physiatrist, two certified GMFM user physiotherapists, two non-certified GMFM user physiotherapists who had experience with childhood disabilities for at least 10 years, and two physiotherapists who had experience with childhood disabilities for less than 5 years; and (h) approving the measure.

### 2.5. Statistical Analysis

Statistical analyses were performed using SPSS Version 26 for Windows (SPSS Inc., Chicago, IL, USA; 2004) and AMOS 23.0 software. Quantitative variables were described by means and standard deviations and qualitative variables by percent. Minimum-maximum scores of individual items and the total value of the sub-scores were examined for floor or ceiling effect. If more than 15% of participants achieved a minimum/maximum score, the presence of a floor or ceiling effect could be mentioned.

Reliability: Intra-class correlation coefficients (ICC) and 95% confidence intervals (CI) with the two-way random-effect model were used for the intra- and inter-rater reliability analysis of the GMFM-88 subdomains, total, and GMFM-66 total scores. The ICC scores were accepted as follows: very high reliability (ICC > 0.90); high reliability (ICC: 0.70–0.89); moderate validity (ICC: 0.50–0.69); low reliability (ICC: 0.26–0.49); poor reliability (ICC < 0.25) [23]. Internal consistency was measured with Cronbach’s alpha coefficient, which was higher than 70% and considered acceptable [24]. The Bland–Altman method was applied to estimate the measurement bias in reliability analysis.

Data normality distributions were assessed with the Kolmogorov–Smirnov test. Since data were distributed non-normally according to the Kolmogorov–Smirnov test (*p* < 0.01), non-parametric tests (Spearman’s rank correlation) were used for relationships.

Standard error of measurement (SEM) and minimal detectable change (MDC): The estimates of SEM and MDC were calculated, based on the same data from 66 subjects who had been assessed twice. The SEM was calculated using the formula: SEM = SD√(1 − ICC) [25]. The MDC was calculated using the formula: MDC = SEM × 1.65 × √2, where 1.65 derives from the 0.90%CI of no change, and √2 shows two measurements assessing the change [26].

Validity: The construct validity of the scale was assessed by calculating Spearman’s Rho correlation coefficient between each sub-domain and the total score of the GMFM and PEDI-mobility scores. Spearman’s rank correlation was used to assess the relationships between the GMFCS and each of the continuous outcome tools used in the study since GMFCS is ordinal rather than continuous. Correlation coefficient accepted as >0.70 strong, 0.50 < *q* < 0.70 moderate, and *q* < 0.50 weak correlation [27]. Finally, confirmatory factor analysis (CFA) was applied to reveal whether the factor structure of the GMFM-88 scale would be confirmed in our sample. To test the construct validity of the scale, the Amos 23.0 program was used, and CFA was performed with the maximum likelihood estimation technique. Each GMFM sub-domain is determined as an independent factor. Chi-Square Goodness, goodness-of-fit-index (GFI), adjusted-goodness-of-fit-index (AGFI), Normed-Fit-Index (NFI), comparative-fit-index (CFI), Tucker-Lewis-index (TLI), which is the equivalent of non-normed-fit-index (NNFI) in the Amos program, relative-fit-index (RFI), incremental-fit-index (IFI), the root-mean-square-of-the-estimation-errors (RMSEA), and the parsimony-normed-fit-index (PNFI) were performed for CFA in this study. In general, for the GFI, CFI, NFI, RFI, and IFI indices, 0.90 indicates acceptable fit and 0.95 indicates perfect fit [28]. For NNFI, a value of 0.95 is taken as an acceptable fit and a value of 0.97 as a measure of perfect fit. For AGFI, 0.85 indicates acceptable fit and 0.90 indicates perfect fit [29]. For the RMSEA, 0.08 indicates an acceptable fit and 0.05 an excellent fit [30,31] A PNFI fit index above 0.50 indicates acceptable fit [32], and >0.95 indicates perfect fit [33].

## 3. Results

The study enrolled 150 children (82 [54.7%] male, 68 [45.3%] female) in an age range between 5 and18 years (mean 8.82 ± 3.78; median 8.00; minimum 5, maximum 18 years). GMFM-88 is a therapy-based survey; no major revision was needed on the Turkish version. No floor and ceiling effect were observed for the subscales of the Turkish GMFM (Table 1).

Distributions of GMFM-88 sub-domains, total, and GMFM-66 total mean scores according to clinical types of CP and GMFCS levels are detailed in Table 2.

### 3.1. Reliability

Internal Consistency: Cronbach’s alpha coefficient for GMFM-88 sub-domains and total score were ranging between 0.997–1.0; 0.998 for GMFM-66.

Intra-Inter Reliability: ICC values demonstrated excellent intra-rater reliability of the GMFM-88 sub-domains and total score (0.994–0.999) as well as the GMFM-66 total score (0.996). ICC values were excellent for both GMFM-88 sub-domains, total score (0.997–0.999), and GMFM-66 score (1.00). The SEM of the measure was low both for GMFM-88 sub-domains and total score (1.044–1.677) and GMFM-66 (1.471). MDC value between 2.435–5.520 for GMFM-88 sub-domains, total score, and 3.431 for GMFM-66. Detailed GMFM psychometric properties results are summarized in Table 3.

**Figure 1:**
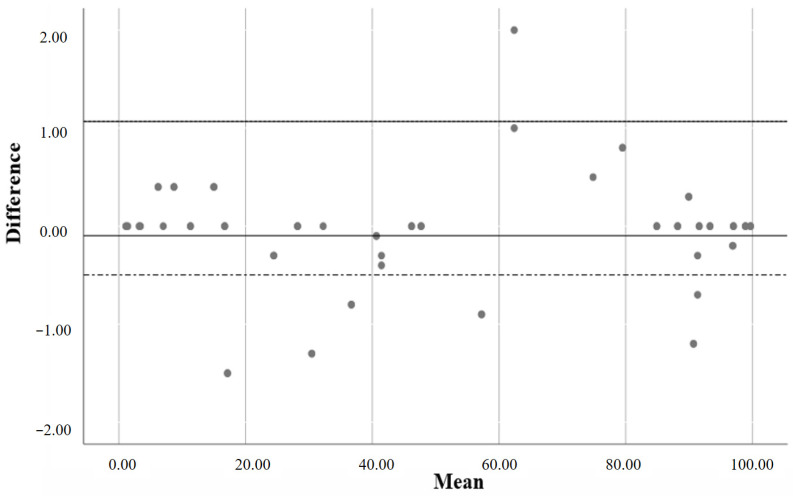
Bland–Altman Graphic for measurement bias to reliability analysis. Legand: ____: The mean value of the difference between two measurements; ------: 95% confidence interval upper bound; – - – - – -: 95% confidence interval lower bound.

According to the Bland–Altman graph, it is seen that the recorded data are largely distributed within the calculated border area, and the existence of bias is calculated as 0.001 (beta: 0.049, *p* = 0.701) (Figure 1).

### 3.2. Validity

Concurrent validity: A strong to excellent negative correlation (*p* < 0.01) found between the GMFCS and the GMFM-88 sub-domains, total score (r: −0.771–−0.955) and GMFM-66 total score (r: −0.736), strong to excellent positive correlation between the PEDI-Mobility and the GMFM-88 sub-domains, total score (r: 0.799–0.988) and GMFM-66 total score (r: 0.968) (Table 4).

Construct Validity: Spearman’s correlation coefficient for between each evaluated domain and GMFM Total score varied between 0.690–0.931. The strongest correlation between GMFM domains was between GMFM-D and GMFM-E (r = 0.931), while the weakest was between GMFM-A and GMFM-C (r = 0.72). (Table 5).

**Scheme 1:**
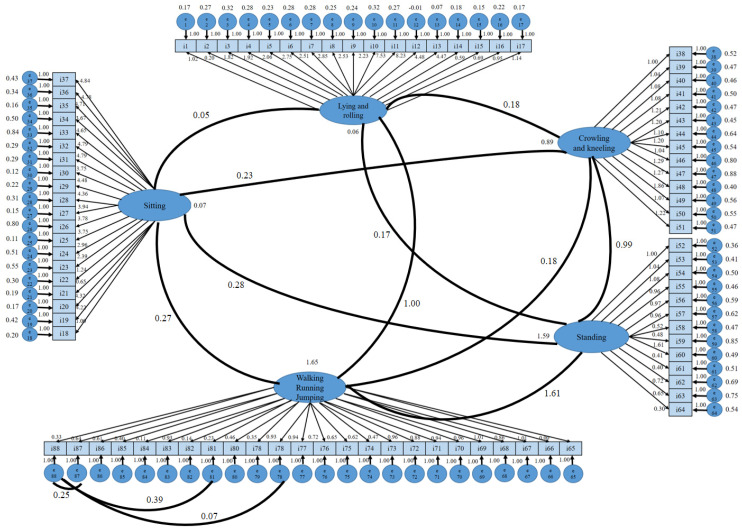
Path diagram of factor analysis.

Structural validity: CFA for the five-dimensional factor structure of the scale, the ratio of chi-square to degrees of freedom (χ^2^ = 559,693; df = 165; *p* = 0.000); RMSEA = 0.084; GFI = 0.848; AGFI = 0.807; CFI = 0.917; NFI = 0.886; TLI = 0.904; RFI = 0.869; IFI = 0.917; It is calculated as RMR = 0.067 and PNFI = 0.77. A model’s comparative fit index (CFI, TLI) height of 0 or above means that it will be in good agreement. It may be accurate in terms of the results obtained (Scheme 1).

## 4. Discussion

GMFM is one of the most frequently used and clinically proven criteria to determine the medical, surgical, and rehabilitation practices of children with CP and the effectiveness of the interventions, as well as to create evidence in clinical CP research [18]. In this current study, we aimed to investigate the inter-rater and intra-rater reliability and validity of the long form, GMFM-88, and the short form, GMFM-66, by creating a Turkish version from the original English version, it was found to be reliable and valid.

In addition to the original English version, the GMFM was translated into many different languages, such as Brazilian Portuguese [12], Persian [5], Thai [34], Korean [13], and recently Spanish in Spain and Colombia [13,15], and their validity and reliability in these languages were investigated. The findings of other studies investigating the psychometric characteristics of cross-cultural adaptations of the GMFM have similar results. For instance, the Brazilian Portuguese version study reported excellent intra- and interrater reliability (ICC = 0.99, 95%CI 0.98–0.99; ICC = 0.97, 95%CI 0.95–0.98) for total scores [12]. The Persian version reported ICC = 0.99 for inter- and intra-rater reliability (95%CI 0.96–1.00) for both total and dimension scores [5]; the Thai study obtained excellent intra- and interrater reliability (ICC = 0.99–1.00; ICC = 0.93) for the total scores [34], results that agree with those obtained by the Korean version, who reported excellent inter- and intra-rater reliability for both total (ICC = 0.99, 95%CI 0.99–0.99) and dimension scores (ICC = 0.97–0.99, 95%CI 0.96–0.99) [13]. The Spanish version of intra- and interrater reliability were excellent for both total (ICC = 1.00, 95%CI 0.99–1.00) and dimension scores (ICC = 0.99, 95%CI 0.99–1.00) [14]. In this study, the intra- and inter-reliability of the Turkish version of the criterion was examined, and the ICC value was found to be 0.998, “perfect” for both GMFM-88 and GMFM-66. Russell et al., in the original version for GMFM-88, reported ICC values as 0.99, that is, “excellent” for intra-rater test, retest, and inter-rater reliability [18]. The high validity and reliability values of almost all languages in which the criterion is translated reflect that the criterion can evaluate the motor functions in children with CP regardless of cultural variables, and thus, it is also important in terms of allowing comparison of individuals with CP living in different communities.

Reliability of a scale is one of the first characteristics examined and refers to the consistency of an assessment tool and the constancy of assessment scores and explains relative or absolute reliability and inter-rater or intra-rater reliability. It has been emphasized that both inter- and intra-rater reliability for this criterion is more important for clinical practice, especially in terms of rehabilitation, since it is required to perform GMFM applications on the same individual many times in order to decide on interventions and determine their effectiveness [18]. Therefore, in our study, the intra-, and inter-reliability of the Turkish version of the criterion was examined, and the ICC value was found to be 0.998, “perfect” for both GMFM-88 and GMFM-66. Russell et al., in the original version for GMFM-88, reported ICC values as 0.99, that is, “excellent” for intra-rater test, retest, and inter-rater reliability [18].

Similar to the original version, Ko and Kim reported an ICC value of 0.95 for intra-observer reliability [35]. This value was found to be higher in our study. Using the video analysis method in the evaluation of test items in order to minimize the disease, fatigue, or environmental factors that may occur between tests and affect the test results in our study, it is thought that minimizing the possible test error may contribute to the occurrence of this difference.

Lewis et al. found a strong significant linear relationship (r = 0.91) between the mobility domain of PEDI-CAT and GMFM-66 scores [36]. Although they did not plan to evaluate the validity of the GMFM, these results provide evidence for the structure validity of the GMFM in terms of mobility. GMFCS, which is used for construct validity, evaluates the mobility level of individuals with CP, and the fact that a high level of significant relationship was found between GMFM sub-dimensions and the total score of GMFM as a result of the study may show similarity in this respect. Ko (2014) investigated the sensitivity of GMFM-88 and GMFM-66, and they investigated the correlation between PEDI-mobility and GMFM-88, GMFM-66, and they found a moderate to strong relationship [37]. Moreover, Lee investigated GMFM and GMFCS and found a strong relationship (r = −0.933), similar to our results [38]. According to all these studies, GMFCS could be used as a golden standard for criterion reference as a parallel test with GMFM, and since it showed an excellent correlation (r = −0.955), it could be said GMFM is a valid measure.

Mehasup et al. reported high intra-observer validity values ranging from 0.99–1 in the validity study of the GMFM-66 Tai version of the criterion [34]. When compared with our study, high values were also achieved in Turkish GMFM-66. However, the number of individuals included in our study (n = 150) seems to be much higher than the Tai study (n = 10).

With the development of the International Classification of Functioning, disability and Health, the focus of CP research has transferred to the field of activity rather than motor impairment, which is also referred to as body structure and functions [1,3]. In this context, GMFCS defines activities related to mobility and movement initiated by the individual with CP and is used quite frequently in the clinical setting [39]. The GMFCS has high validity and reliability in both the original English version and Turkish version. According to Ferre-Fernández (2022), 86% of the total score of the GMFM-SP-88 may be explained by the GMFCS level [14]. Therefore, in the validity assessment of this study, the criterion of Turkish validity and reliability GMFCS was used, and a strong to excellent negative correlation was found between Turkish GMFM-88 and its sub-dimensions and the GMFM-66. Park et al. (2005) found a strong negative relationship between GMFM-88 and GMFCS similar to our study [13]. In this current study, it is important in this sense that the strongest relationship between GMFCS and GMFM-88 sub-dimensions is with the walking, running, and jumping sub-dimensions.

In addition to correlation analysis with GMFCS and PEDI-Mobility, construct validity was analyzed using factor analysis and in our study. The most frequently used method to determine the validity of using a scale in a different culture is CFA [40]. The measurements of the structures in the model were examined for the correct measurements of the relevant structures, and the structural models were separated from each other. In CFA, each subdomain—lying and rolling, sitting, crawling and kneeling, standing and walking/running/jumping—were accepted as separate factors, and the relationship as well as distribution of items of GMFM were investigated with CFA. The CFA confirmed that Turkish GMFM is fitted for the five-dimensional factor structure. In this current study, part of the measurement error of one indicator is partially correlated with the measurement error of another indicator. Hermida explained the reasons for correlated errors. There is evidence that there exists a cause of both of the variables to which the residuals are attached, but that is not specified in the model [41]. Therefore, part of the measurement error of one indicator is partially correlated with the measurement error of another indicator. This correlation could come from pure randomness or from something that influences both indicators. Motor development includes the evolution from basic movements and goal-directed motor actions. These motor actions are never performed in isolation, often requiring interaction. For a child to function within this context, they require the ability to demonstrate skillful, efficient, and voluntary postures and movement patterns [42]. Therefore, as the developers of original the GMFM indicate, for the total measurement, the relation between each subdomain is also investigated since motor function itself has importance and each subdomain contributes to motor function development [18]. This study is one of the studies investigating factor analysis. On the other hand, the GMFM on itself has 88 items, and therefore, the number of the children with CP was constrained for fit index. Therefore, it is needed to analyze the fit index with a larger sample size for future studies.

The main limitation of this study is that, although it evaluated a wide range of individuals, young children were not included. Another important limitation is the lower proportion of children with severe motor function impairments, classified in GMFCS level V, especially in the ataxic subtype; this point can limit result generalization. Therefore, further studies are needed to examine validity and reliability among observers and to expand the age range of the individuals as well as with different GMFCS levels of different subtypes of CP. Additionally, since the data of this study do not follow a normal distribution, for further analysis, CFA needs a larger sample size; in future studies with a larger sample size, CFA needs to be tested.

## 5. Conclusions

In conclusion, according to the results, both Turkish versions of GMFM-88 and GMFM-66 are valid and reliable measures in evaluation of the gross motor function of children with CP in the age group 5–18, and the GMFM-88 and GMFM-66 are consistent in determining the gross motor function levels.

## Figures and Tables

**Table 1:** Demographic characteristics and clinical data of participants at baseline (n = 150).

Age, (Range) Mean (Standard Deviation), Median: (5–18 y), 8.82 (3.78), 8.00							
Gender, n (%)	Male	82 (54.7)					
	Female	68 (45.3)					
GMFCS Levels, n (%)	I	30 (20.00)					
	II	30 (20.00)					
	III	30 (20.00)					
	IV	30 (20.00)					
	V	30 (20.00)					
		Age mean (Standard deviation)	GMFCS I	GMFCS II	GMFCS III	GMFCS IV	GMFCS V
Clinical Type, n (%)	Spastic 107 (71.33)	8.68 (3.55)	30	20	25	13	19
	Dyskinetic 35 (23.33)	9.08 (4.61)	-	4	3	17	11
	Ataxic 8 (5.33)	9.00 (2.91)	1	5	2	-	-
Centre of origin, n (%)	Special education and rehabilitation center	105 (70)					
	University hospital	30 (20)					
	Private practice	15 (10)					
GMFM			Mean	Standard deviation	Median	Minimum	Maximum
GMFM-88-Lying and rolling			81.86	27.59	96.10	2.00	100.00
GMFM-88-Sitting			66.14	34.29	78.30	0.00	100.00
GMFM-88-Crawling and kneeling			53.30	40.59	60.70	0.00	100.00
GMFM-88-Standing			34.35	36.81	20.50	0.00	100.00
GMFM-88-Walking/running/jumping			32.25	35.61	18.00	0.00	98.60
GMFM-88-Total Score			53.58	31.89	55.51	2.00	99.72
GMFM-66 Score			49.74	22.40	48.50	4.10	100.00
GMFM-88-Lying and rolling-re-test			76.96	28.98	93.00	3.00	99.00
GMFM-88-Sitting re-test			62.76	34.66	68.30	4.00	99.00
GMFM-88-Crawling and kneeling-re-test			45.27	41.89	40.50	0.00	99.00
GMFM-88-Standing-re-test			35.42	38.20	15.00	0.00	100.00
GMFM-88-Walking/running/jumping-re-test			34.40	38.35	5.20	0.00	96.00
GMFM-88-Total Score re-test			51.37	34.14	46.96	1.40	98.40
GMFM-66 Score re-test			49.00	24.10	44.50	8.00	99.00
GMFM 88 Total Scores According to the GMFCS Levels							
GMFCS Level I			64.614	4.441	96.290	84.38	99.72
GMFCS Level II			75.78	11.23	79.33	46.52	90.04
GMFCS Level III			57.96	11.86	56.34	39.84	87.86
GMFCS Level IV			29.00	9.99	28.20	8.94	47.74
GMFCS Level V			10.55	5.37	11.38	2.00	17.28
Pediatric Evaluation of Disability Inventory							
Pediatric Evaluation of Disability Inventory— Mobility			53.64	32.60	54.23	0.00	100.00

GMFM, Gross Motor Function Measurement; GMFCS, Gross Motor Function Classification System; n, number; %, per cent.

**Table 2:** Distributions of GMFM scores according to clinical types and GMFCS levels.

GMFM	GMFCS I			GMFCS II			GMFCS III			GMFCS IV			GMFCS V		
	Spas.	Dys.	At.	Spas.	Dys.	At.	Spas.	Dys.	At.	Spas.	Dys.	At.	Spas.	Dys.	At.
	X (SD)	X ± SD	X ± SD	X ± SD	X ± SD	X ± SD	X ± SD	X ± SD	X ± SD	X ± SD	X ± SD	X ± SD	X ± SD	X ± SD	X ± SD
A	98.90 ± 1.53	-	-	97.87 ± 2.17	96.10 ± 0	100 ± 0	98.58 ± 1.95	86.66 ± 4.09	100 ± 0	78.26 ± 20.36	83.18 ± 9.93	-	39.65 ± 20.01	23.70 ± 19.56	-
B	98.03 ± 2.31	-	-	94.69 ± 5.09	85.0 ± 7.73	98.33 ± 1.47	84.01 ± 11.62	71.10 ± 12.47	80.85 ± 8.27	40.51 ± 19.64	40.58 ± 16.58	-	15.17 ± 9.29	16.05 ± 8.34	-
C	97.61 ± 4.29	-	-	86.80 ± 17.33	79.80 ± 1.38	80.16 ± 14.18	64.87 ± 23.92	48.40 ± 5.54	80.95 ± 16.89	23.44 ± 21.75	15.55 ± 21.71	-	1.51 ± 2.14	0.43 ± 1.26	-
D	90.76 ± 12.12	-	-	53.97 ± 19.66	33.30 ± 23.67	63.26 ± 23.46	25.33 ± 19.56	15.40 ± 13.33	29.50 ± 12.72	3.54 ± 5.69	3.37 ± 2.95	-	1.42 ± 1.64	0.81 ± 0.60	-
E	87.76 ± 9.29	-	-	52.44 ± 22.26	37.50 ± 17.66	45.83 ± 16.14	23.22 ± 21.81	13.60 ± 1.97	23.60 ± 1.97	1.92 ± 3.27	0.30 ± 1.23	-	0.63 ± 0.89	0.43 ± 0.97	-
GMFM-88 Total	94.61 ± 4.44	-	-	77.15 ± 11.95	66.34 ± 10.09	77.52 ± 6.72	59.20 ± 11.81	44.31 ± 3.23	62.98 ± 7.97	29.54 ± 12.88	28.60 ± 7.49	-	11.67 ± 5.30	8.60 ± 5.15	-
GMFM-66 Total	80.98 ± 9.55	-	-	61.69 ± 7.56	55.35 ± 7.10	58.43 ± 4.13	50.38 ± 6.72	43.73 ± 1.15	70.75 ± 30.19	38.09 ± 11.12	35.22 ± 5.63	-	20.32 ± 6.93	19.92 ± 6.11	-

A, Lying and rolling; At., Ataxic; B, Sitting; C, Crawling and kneeling; D, Standing; Dys, Dyskinetic; E, Walking/running/jumping; GMFM, Gross Motor Function Measurement; GMFCS, Gross Motor Function Classification System; SD, Standard Deviation; Spas., Spastic; X, Mean.

**Table 3:** Reliability Summary Statistics of GMFM.

GMFM Domains	Internal Consistency Alpha ^a^	Inter-Rater Reliability			Intra-Rater Reliability			SEM	MDC90
		ICC	95%CI	*p*	ICC	95%CI	*p*		
A. Lying and rolling	0.997	0.997	0.996–0.998	<0.001	0.994	0.990–0.996	<0.001	1.549	5.520
B. Sitting	0.999	0.998	0.997–0.998	<0.001	0.998	0.996–0.999	<0.001	1.541	3.595
C. Crawling and kneeling	0.999	0.997	0.996–0.998	<0.001	0.999	0.998–0.999	<0.001	1.304	3.042
D. Standing	0.998	0.994	0.991–0.995	<0.001	0.998	0.996–0.999	<0.001	1.677	3.912
E. Walking/running/ jumping	0.999	0.997	0.996–0.998	<0.001	0.999	0.998–0.999	<0.001	1.216	4.339
GMFM-88 Total	1.00	0.999	0.999–0.999	<0.001	0.999	0.999–1.00	<0.001	1.044	2.435
GMFM-66 Total	0.998	1.00	1.00–1.00	<0.001	0.996	0.994–0.998	<0.001	1.471	3.431
GMFCS Level I	0.964	0.942	0.881–0.972	<0.001	0.931	0.814–0.975	<0.001	0.756	1.736
GMFCS Level II	0.997	0.988	0.976–0.994	<0.001	0.995	0.980–0.999	<0.001	0.481	1.122
GMFCS Level III	0.983	0.995	0.989–0.997	<0.001	0.966	0.780–0.995	<0.001	1.076	2.510
GMFCS Level IV	0.999	0.995	0.990–0.998	<0.001	0.998	0.994–0.999	<0.001	0.974	2.272
GMFCS Level V	0.996	0.988	0.975–0.994	<0.001	0.991	0.975–0.997	<0.001	1.052	2.454

^a^, Cronbach’s alpha; CI, confidence interval; GMFCS, gross motor function classification system; GMFM, gross motor function measurement; ICC, interclass correlation coefficient; MDC, minimal detectible change; *p*, *p* value; SEM, standard error of measurement.

**Table 4:** Correlations between GMFM, GMFCS and PEDI-Mobility.

		GMFM-88 Lying and Rolling	GMFM-88 Sitting	GMFM-88 Crawling and Kneeling	GMFM-88 Standing	GMFM-88 Walking/ Running/ Jumping	GMFM-88 Total	GMFM-66 Total
GMFCS	Spearman rho	−0.771	−0.901	−0.895	−0.868	−0.887	−0.955	−0.736
	*p*	<0.01	<0.01	<0.01	<0.01	<0.01	<0.01	<0.01
	n	150	150	150	150	150	150	150
PEDI Mobility	Spearman rho	0.799	0.930	0.939	0.899	0.925	0.988	0.968
	*p*	<0.01	<0.01	<0.01	<0.01	<0.01	<0.01	<0.01
	n	150	150	150	150	150	150	150

GMFM, Gross Motor Function Measurement; GMFCS, Gross Motor Function Classification System.

**Table 5:** Correlations between sub-domains and total score of GMFM-88.

		GMFM-88 Lying and Rolling	GMFM-88 Sitting	GMFM-88 Crawling and Kneeling	GMFM-88 Standing	GMFM-88 Walking/Running/ Jumping
GMFM-88 Sitting	r	0.853				
	*p*	<0.01				
	n	150				
GMFM-88 Crawling and kneeling	r	0.790	0.890			
	*p*	0.001	<0.01			
	n	150	150			
GMFM-88 Standing	r	0.690	0.843	0.866		
	*p*	<0.01	<0.01	<0.01		
	n	150	150	150		
GMFM-88 Walking/running/ jumping	r	0.729	0.864	0.889	0.931	
	*p*	<0.01	<0.01	<0.01	<0.01	
	n	150	150	150	150	
GMFM-88 Total	r	0.817	0.937	0.951	0.904	0.934
	*p*	<0.01	<0.01	<0.01	<0.01	<0.01
	n	150	150	150	150	150

## Data Availability

Data is unavailable due to privacy.
